# Flexible sensors with zero Poisson's ratio

**DOI:** 10.1093/nsr/nwae027

**Published:** 2024-01-23

**Authors:** Xin Huang, Tianzhao Bu, Qingyang Zheng, Shaoyu Liu, Yangyang Li, Han Fang, Yuqi Qiu, Bin Xie, Zhouping Yin, Hao Wu

**Affiliations:** Department of Mechanical Engineering, Flexible Electronics Research Center, State Key Laboratory of Intelligent Manufacturing Equipment and Technology, School of Mechanical Science and Engineering, Huazhong University of Science and Technology, Wuhan 430074, China; Department of Mechanical Engineering, Flexible Electronics Research Center, State Key Laboratory of Intelligent Manufacturing Equipment and Technology, School of Mechanical Science and Engineering, Huazhong University of Science and Technology, Wuhan 430074, China; Department of Mechanical Engineering, Flexible Electronics Research Center, State Key Laboratory of Intelligent Manufacturing Equipment and Technology, School of Mechanical Science and Engineering, Huazhong University of Science and Technology, Wuhan 430074, China; Department of Mechanical Engineering, Flexible Electronics Research Center, State Key Laboratory of Intelligent Manufacturing Equipment and Technology, School of Mechanical Science and Engineering, Huazhong University of Science and Technology, Wuhan 430074, China; Department of Mechanical Engineering, Flexible Electronics Research Center, State Key Laboratory of Intelligent Manufacturing Equipment and Technology, School of Mechanical Science and Engineering, Huazhong University of Science and Technology, Wuhan 430074, China; Department of Mechanical Engineering, Flexible Electronics Research Center, State Key Laboratory of Intelligent Manufacturing Equipment and Technology, School of Mechanical Science and Engineering, Huazhong University of Science and Technology, Wuhan 430074, China; Department of Mechanical Engineering, Flexible Electronics Research Center, State Key Laboratory of Intelligent Manufacturing Equipment and Technology, School of Mechanical Science and Engineering, Huazhong University of Science and Technology, Wuhan 430074, China; Department of Mechanical Engineering, Flexible Electronics Research Center, State Key Laboratory of Intelligent Manufacturing Equipment and Technology, School of Mechanical Science and Engineering, Huazhong University of Science and Technology, Wuhan 430074, China; Department of Mechanical Engineering, Flexible Electronics Research Center, State Key Laboratory of Intelligent Manufacturing Equipment and Technology, School of Mechanical Science and Engineering, Huazhong University of Science and Technology, Wuhan 430074, China; Department of Mechanical Engineering, Flexible Electronics Research Center, State Key Laboratory of Intelligent Manufacturing Equipment and Technology, School of Mechanical Science and Engineering, Huazhong University of Science and Technology, Wuhan 430074, China; Department of Electronic Science and Technology, School of Integrated Circuits, Huazhong University of Science and Technology, Wuhan 430074, China

**Keywords:** zero Poisson's ratio, metamaterials, independent perception of multiaxial stimuli, flexible sensor

## Abstract

Flexible sensors have been developed for the perception of various stimuli. However, complex deformation, usually resulting from forces or strains from multi-axes, can be challenging to measure due to the lack of independent perception of multiaxial stimuli. Herein, flexible sensors based on the metamaterial membrane with zero Poisson's ratio (ZPR) are proposed to achieve independent detection of biaxial stimuli. By deliberately designing the geometric dimensions and arrangement parameters of elements, the Poisson's ratio of an elastomer membrane can be modulated from negative to positive, and the ZPR membrane can maintain a constant transverse dimension under longitudinal stimuli. Due to the accurate monitoring of grasping force by ZPR sensors that are insensitive to curvatures of contact surfaces, rigid robotic manipulators can be guided to safely grasp deformable objects. Meanwhile, the ZPR sensor can also precisely distinguish different states of manipulators. When ZPR sensors are attached to a thermal-actuation soft robot, they can accurately detect the moving distance and direction. This work presents a new strategy for independent biaxial stimuli perception through the design of mechanical metamaterials, and may inspire the future development of advanced flexible sensors for healthcare, human–machine interfaces and robotic tactile sensing.

## Teaser

This paper presents a generic strategy to alter the Poisson's ratio of elastomer membranes, and achieve flexible sensors with zero Poisson's ratio, which have the capability of independently detecting biaxial stimuli.

## INTRODUCTION

Flexible sensors have gained many exotic sensing capabilities in the past decade, which impressively push the frontiers of healthcare, human–machine interfaces and robotic perception [1–6]. Numerous innovative attempts have been made to improve the sensitivity, measurement range and response time of flexible sensors [7–12]. Although flexible sensors have demonstrated reliability when measuring uniaxial external stimuli, their perception capability still has a critical limitation when external stimuli are multiaxial [13,14]. For instance, the complex deformation of grasped objects or human skin from multi-axes dramatically compromises the measurement accuracy of flexible sensors, leading to occurrences of unstable grasping states or erroneous physiological signals. Due to the inherent Poisson's ratio of sensing materials, stimuli applied in a particular axis will induce corresponding deformation in the perpendicular axis, leading to interference signals in detection of stimuli in the perpendicular axis [15]. The Poisson's effect of sensing materials is the main obstacle for independent perception of biaxial stimuli. Notably, flexible sensors with independent perception of multiaxial stimuli can tackle the challenges imposed by those scenarios. Although elaborately arranged functional materials can detect uniaxial stimuli without interference, the stimuli from the other perpendicular-axis stimuli cannot be measured [16,17]. Multiaxial flexible sensors capable of measuring multiaxial stimuli were also reported, but the response signals to multiaxial stimuli were not fully decoupled and complex data processing was required to extract the responses of different stimuli [18,19]. Thus, independent perception of multiaxial stimuli remains a great challenge for flexible sensors.

Zero Poisson's ratio (ZPR) materials, which maintain constant transverse width under longitudinal strain, can effectively address the interference issues of flexible sensors in biaxial or multiaxial stimuli perception. However, while a few materials, including glasses, minerals and corks, exhibit Poisson's ratio of near zero [20,21], none of these materials can be utilized to fabricate flexible sensors. With the elaborate engineering of the structure and the careful arrangement of unit cells, metamaterials have achieved non-natural and oftentimes counterintuitive characteristics [22–27]. In particular, mechanical metamaterials, as a subgroup of metamaterials, have the ability to dramatically alter the mechanical behavior of materials through distinct design of geometry, which provides great potential for realizing ZPR [28–33]. Extensive efforts have been made to create structural design approaches for altering the Poisson's ratio of materials with rigid elements, such as the creation of auxetic foam based on conventional foam [34–36], construction of auxetic honeycomb with rigid elements and flexible hinges [37–41], an origami structure with predefined crease patterns [42–46] and kirigami-based materials with elaborate arrays of cuts [47–50]. However, there was limited research on a structural design of stretchable elastomer that would achieve ZPR. Compared with rigid elements in mechanical metamaterials with lattice structures, the stretchable elastomer elements are prone to elongation or contraction rather than rotation under external stimuli [51], which increases the influence of the Poisson's ratio of constituent materials to the Poisson's ratio of the overall structure. Since the elastomer is usually considered incompressible and exhibits a Poisson's ratio of nearly 0.5, both elaborate geometry design and meticulous arrangement of elements are required to achieve ZPR.

In this study, we propose ZPR-membranes-enabled flexible sensors capable of independently detecting biaxial stimuli. The membrane's structure is designed and optimized based on the combination of positive and negative Poisson's ratio structures. Using gold thin film for the active conductive layers on elaborately designed sensing areas, the ZPR sensor is capable of accurately detecting biaxial strain without any interference. We demonstrate that the unique characteristics of the ZPR flexible sensors enable accurate force, strain and motion perception in cases of robotic manipulation and locomotion with complex deformation, while sensors without ZPR provide erroneous measurements. Specifically, we demonstrate that different curvatures hardly affect the signal of the ZPR sensor perpendicular to the circumference, which enables the ZPR sensor to accurately measure contact forces and guide rigid manipulators to grasp deformable objects. Uniaxial bending has no effect on sensing areas perpendicular to the bending direction, allowing the ZPR sensor attached on the surface of manipulators to monitor the occurrence of collisions during manipulation tasks. Moreover, a thermal-actuation soft robot capable of walking independently on two axes is utilized to evaluate the performance of the ZPR sensor, which demonstrates that the walking direction and distance can be distinctly perceived. The ZPR-membrane-enabled flexible sensors by mechanical metamaterials achieve independent detection of biaxial stimuli, which may facilitate the development of tactile perception systems and the realization of proprioceptive soft robots.

## RESULTS AND DISCUSSION

### Design of ZPR structure

**Figure 1.**
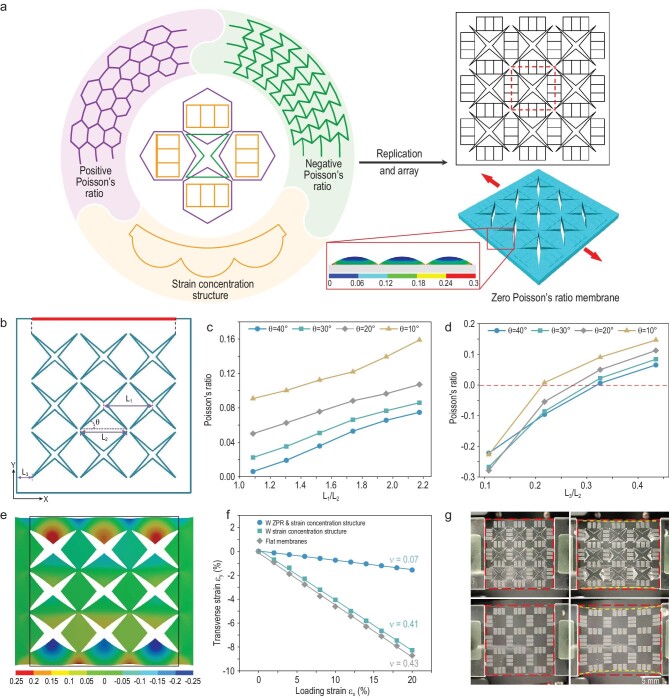
Structural design for ZPR. (a) The design principle of the ZPR structure is conceived by combining concave structures and hexagonal structures in an elaborately designed position, while the strain concentration structure is embedded in the middle of the hexagonal structures. The dotted wireframe represents a single element of the ZPR structure. The inset in the wireframe illustrates local strain distribution of the strain concentration structure under uniaxial stretching, showing that the bottom of the wavy structure has the highest tensile strain. (b) The geometry of the ZPR structure, the longitudinal deformations of the boundary highlighted by the red line under transverse strain, are utilized to calculate the Poisson's ratio. (c and d) Poisson's ratio of membranes with different concave angle θ, different L_1_ (c) and different L_3_ (d) obtained by FEA. (e) A longitudinal deformation contour map of the membrane (L_1_/L_2_ = 1.1, L_3_/L_2_ = 0.3, θ = 40°) under 20% transverse tensile strain. The black wireframe represents the initial size of the membrane. (f) The Poisson's ratio and transverse strain, measured by analyzing the images during the stretching process, of membranes with a ZPR structure and strain concentration structure, membranes with only a strain concentration structure, and flat membranes under 20% longitudinal tensile strain. (g) Photographs of membranes with the structure (top) and with only a strain concentration structure (bottom) under 0% tensile strain (left) and 20% tensile strain (right).

The Poisson's ratio of the mechanical metamaterial is closely related to element geometric structures, the arrangement of elements and the Poisson's ratio of the constituting materials. By merging the hexagonal structure of positive Poisson's ratio (PPR) materials and the concave structure of negative Poisson's ratio (NPR) materials, the Poisson's ratio of the hybrid structure is the superposition of these structures’ Poisson's ratios. It is possible to realize ZPR of the hybrid structure by optimizing parameters and elaborately selecting constituting materials. As shown in Fig. 1a, a single element of the ZPR structure consists of four hexagonal structures and one concave structure. The hexagonal structures are uniformly distributed around the concave structure, which ensures the central symmetry and the same Poisson's ratio on both axes. Notably, the concave structure is hollow, while the surrounding hexagonal structures are filled with the substrate material. Moreover, strain concentration structures are introduced in the middle of the hexagonal structure to optimize sensing performance. After replication and array, nine elements compose the entire membrane, with adjacent elements sharing the same hexagonal structure. Due to its remarkable stretchability and facile preparation process, polydimethylsiloxane (PDMS) was utilized as the substrate material in this work. To calculate the Poisson's ratio of the hybrid structure, the displacements of boundaries under uniaxial strain need to be determined. The deformation of membranes with the hybrid structure under uniaxial strain can be described by the Cauchy equation [52]:


(1) \begin{eqnarray*}
{{\mathrm{\varepsilon }}}_{{i}} &&= \frac{{\partial {{{u}}}_{{i}}}}{{\partial {{i}}}}{\mathrm{,\ }}\quad{{\mathrm{\gamma }}}_{{{ij}}} = {{\mathrm{\gamma }}}_{{{ji}}} = \frac{{\partial {{{u}}}_{{i}}}} {{\partial {{\!j}}}} + \frac{{\partial {{{u}}}_{{j}}}}{{\partial {{i}}}}\nonumber\\
&&\quad\quad \quad {{\ i\ }} \ne {{\ j\quad and\quad i,\!j\ = \ x,y,z}},
\end{eqnarray*}

where *ε* is the normal strain, *γ* is the shear strain and *u* is the displacement. Meanwhile, the elastomer membrane should satisfy the equilibrium condition:


(2) \begin{eqnarray*}
&& \frac{{\partial {{\mathrm{\sigma }}}_{{i}}}} {{\partial {{i}}}} + \frac{{\partial {{\mathrm{\tau }}}_{{{ji}}}}}{{\partial {{\!j}}}} + \frac{{\partial {{\mathrm{\tau }}}_{{{ki}}}}}{{\partial {{k}}}} + {{{f}}}_{{i}} = {\mathrm{ 0, }} \nonumber\\
&& \quad{{\mathrm{\tau }}}_{{{ij}}} = {{\mathrm{\tau }}}_{{{ji}}},{{\ i\ }} \ne {{\ j }} \ne {{\ k \quad {\rm and \quad }}} {{i,j,k\ = \ x,y,z}}, \nonumber\\
\end{eqnarray*}

where *σ* represents the normal stress, *τ* represents the shear stress and *f* represents the body force. Furthermore, the membrane follows the constitutive equation:


(3) \begin{eqnarray*}
{\sigma}_{{i}} &=& { \lambda } \left( {\varepsilon }_{{i}}+ \varepsilon_{{j}} + \varepsilon_{{k}} \right) + 2G \varepsilon_{{i}},\quad {\tau }_{{{jk}}} = {{G}}{\gamma}_{{{jk}}}\nonumber\\
&& {{\ i\ }} \ne {{\ j\ }} \ne {\ k} \quad{\rm and}\quad {{ i,j,k}}\ = \ {{x,y,z}},
\end{eqnarray*}

where *λ* and *G* are Lame constants. As we are dealing with displacement, equations (1)–(3) can be solved by the displacement method and are converted to the Navier equation:


(4) \begin{eqnarray*}
&&\left( {{{\lambda + G}}} \right)\frac{\partial }{{\partial {{i}}}}\left( {\frac{{\partial {{{u}}}_{{i}}}} {{\partial {{i}}}} + \frac{{\partial {{{u}}}_{{j}}}}{{\partial {{j}}}} + \frac{{\partial {{{u}}}_{{k}}}}{{\partial {{k}}}}} \right)\nonumber\\
&& \quad +{{ G}} \left(\frac{{{\partial }^{\mathrm{2}}{{{u}}}_{{i}}}}{{\partial {{{i}}}^{\mathrm{2}}}} + \frac{{{\partial }^{\mathrm{2}}{{{u}}}_{{i}}}}{{\partial {{{j}}}^{\mathrm{2}}}} + \frac{{{\partial }^{\mathrm{2}}{{{u}}}_{{i}}}}{{\partial {{{k}}}^{\mathrm{2}}}} \right) + {{{f}}}_{{i}} = 0 \nonumber\\
&&{{ \ i\ }} \ne {{\ j\ }} \ne {{\ k}} \quad {\mathrm{and}}\quad {{i,j,k}} = {{x,y,z}},
\end{eqnarray*}

as the thickness of the membrane with the hybrid structure is much less than the structure dimensions. Equation (4) can be transformed into planar form, and the boundary condition can be defined as:


(5) \begin{equation*}{u}_x = \overline {{u}_x}
\end{equation*}

(6) \begin{eqnarray*}
&& G\frac{\partial }{{\partial x}}\left( {\frac{{\partial {u}_x}}{{\partial y}} + \frac{{\partial {u}_y}}{{\partial x}}} \right) + \frac{\partial }{{\partial y}}\Bigg[ \lambda \left( {\frac{{\partial {u}_x}}{{\partial x}} + \frac{{\partial {u}_y}}{{\partial y}}} \right)\nonumber\\
&&\quad\quad +\, 2G\frac{{\partial {u}_y}}{{\partial y}} \Bigg] = 0.
\end{eqnarray*}

For the given displacement of the X-axis boundary (*u_x_*), the displacement of the Y-axis boundary (*u_y_*) can be calculated by solving equations (5) and (6). With both *u_x_* and *u_y_*, the Poisson's ratio of the overall structure can be obtained. Since the Lame constants (*G* and *λ*) are determined by the material properties and structural dimensions of the membrane with the hybrid structure, the optimum design of structural dimensions can reduce the displacement of the Y-axis boundary and realize ZPR. To simplify the sophisticated calculation in the process of optimizing Poisson's ratio, finite element analysis (FEA) was utilized to estimate the Poisson's ratio of the hybrid structure. The feature size of PPR and NPR structures represents the proportion of PPR and NPR on the overall Poisson's ratio, and the change of feature size and width of the hybrid structure can vary the Poisson's ratio between positive and negative. Parameters of the hybrid structure were optimized to obtain a Poisson's ratio of almost zero. As shown in Fig. 1b, the characteristic length of the concave structure (*L*_2_) is fixed to a constant, while the concave angle (*θ*), the characteristic length of the hexagonal structure (*L*_1_), and the distance between the concave structures and edges of the membrane (*L*_3_) can be varied. The ratio of *L*_1_ and *L*_2_ and the ratio of *L*_3_ and *L*_2_ were regarded as optimized parameters instead of *L*_1_ and *L*_3_. The deformation of the longitudinal boundaries of membranes with different parameters under 20% lateral strain were recorded (Figs S1 and S2).

The Poisson's ratio of membranes with the same *L*_3_/*L_2_* of 0.3, different *L*_1_*/L_2_* and *θ* were analyzed at first. The result shown in Fig. 1c indicates that the Poisson's ratio of the membrane increases with the increase of *L*_1_*/L_2_* and the decrease of *θ*, and achieves almost zero under *L*_1_*/L_2_* of 1.1 and *θ* of 40°. Since *L*_2_ remains constant, an increase in *L*_1_ is equivalent to the large proportion of PPR, resulting in the increase of the overall Poisson's ratio. On the other hand, the decrease of *θ* attenuates the concave effect of the structure, which reduces the proportion of NPR and increases the overall Poisson's ratio. Since the membrane area within *L*_3_ is flat without any structure, the increase of *L*_3_ also manifests the increase in the proportion of PPR and the overall Poisson's ratio, which is demonstrated by the result shown in Fig. 1d. The membrane with *L*_3_/*L_2_* of 0.3 exhibits a Poisson's ratio closest to zero. In particular, when *L*_3_/*L_2_* is reduced to <0.3, the overall Poisson's ratio of the membrane converts to NPR, and increases with the decrease of *L*_3_. However, the decrease of *L*_3_ apparently increases the local strain in corner areas of the concave structure near the boundary, which weakens the mechanical stability of the membrane. Generally, the membrane with *L*_1_*/L_2_* of 1.1, *L*_3_/*L_2_* of 0.3 and *θ* of 40° exhibits an optimal Poisson's ratio of almost zero. The longitudinal deformation of the optimized membrane under 20% transverse tensile strain is shown in Fig. 1e, and the black border indicates the initial membrane boundary. The strain concentration structures have altered the local strain distribution of hexagonal areas (Fig. S3). When hexagonal areas are covered with conductive materials, strain concentration structures can induce cracks on specific positions under external stimuli, which contributes to more stable sensing signals compared to flat surfaces with scattered channel cracks. The effect of a strain concentration structure on the Poisson's ratio was also analyzed. The membrane with a strain concentration structure exhibits a similar absolute value of deformation along the longitudinal boundary (Fig. S4), resulting in a similar Poisson's ratio. The strain contour map of membranes with and without strain concentration structures further demonstrates that the strain concentration structure decreases the local strain in hexagonal structures, which contributes to extending the sensing range of the sensors with ZPR membranes (Fig. S5).

Three types of membranes were prepared to confirm the unique Poisson's ratio of the structure, and verify the effect of different structures. As shown in Fig. 1f, flat membranes, and membranes with only a strain concentration structure, exhibit similar Poisson's ratios, while membranes with a ZPR structure and strain concentration structure present a small Poisson's ratio of 0.07, indicating that the combination of hexagonal and concave structures is the major contributor to the decrease in Poisson's ratio. The discrepancy of the Poisson's ratio between actual stretching and simulation analysis is mainly due to the difficulty in accurately controlling the dimensions during fabrication processes and the different material properties in experiment and simulation. The PDMS membrane with a ZPR structure exhibits nearly constant length in the direction perpendicular to the loading strain under 10% uniaxial tensile strain, while the membrane without a ZPR structure shows a clear decrease in the longitudinal length (Fig. 1g and Movie S1). The overall mechanical properties of membranes with the structure were also measured (Fig. S6). Compared to flat membranes, the membrane with a ZPR structure and strain concentration structure has small modulus due to the concave holes.

### Fabrication of ZPR-metamaterial-based strain sensors

**Figure 2.**
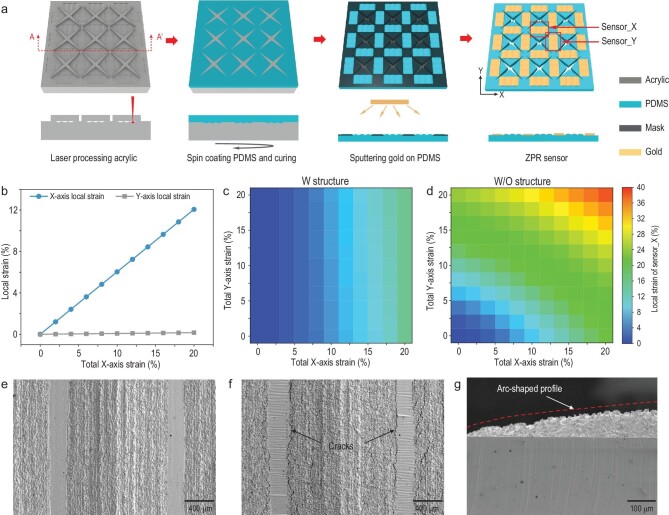
The biaxial independent perception mechanism of ZPR sensors. (a) Schematic illustration of the fabrication process of ZPR sensors. (b) Local strain of sensing units under uniaxial X-axis tensile strain. (c and d) Local strain of the X-axis sensing unit on membranes with a ZPR structure and strain concentration structure (c), and with only a strain concentration structure (d) under different biaxial strains. (e and f) SEM images of the X-axis sensing unit before (e) and after (f) uniaxial X-axis stretching. (g) Cross-sectional SEM image of half of the arc-shaped profile of the strain concentration structure.

The fabrication processes of ZPR sensors are schematically shown in Fig. 2a. The laser engraving process was first utilized to prepare wavy grooves in specific areas, which became regions with strain concentration structures after PDMS molding. Then, the star-shaped acrylic sheets prepared by laser cutting were stuck on specific locations of the acrylic surface. After preparation of the acrylic mold, PDMS was poured into the mold and spin coated to obtain membranes with specific thicknesses. Based on strain distribution of the membrane with a ZPR structure (Fig. S5), the conductive layer was coated on the sensing areas with a strain concentration structure to obtain stable signals. Due to outstanding sensitivity, stability and a facile fabrication process, gold thin film was chosen as the conductive layer. The membrane surface was covered with a mask to ensure that gold was only deposited on the surface of the sensing units, where the strain concentration structure was located. Due to the ZPR characteristic, the electric resistance of the gold layer on the X-axis and Y-axis sensing unit is correlated with mechanical stimuli only in X-axis or Y-axis, respectively. To analyze the mechanism of independent perception of biaxial strain by ZPR sensors, FEA was utilized to calculate the local strain of sensing units under the biaxial strain condition.

At first, the X-axis uniaxial tensile strain was applied to the ZPR membrane, while the Y-axis was free of load. The local strain of the X-axis and Y-axis sensing units (sensor_X and sensor_Y) obtained by FEA is shown in Fig. 2b. Sensor_Y exhibits a very low level of local strain under X-axis uniaxial tensile strain, while sensor_X shows strain value equal to the applied strain. The local strain under only Y-axis tensile strain shows similar results (Fig. S7). Moreover, the local strain of sensing units on the membranes with and without a ZPR structure under different biaxial strains were analyzed to demonstrate the independent perception of biaxial stimuli by the sensor with a ZPR structure. The analysis results are exhibited in Fig. 2c and d. The horizontal and vertical axes of the contour map indicate X-axis and Y-axis tensile strain applied to the membrane, while the contour map represents the local strain of sensor_X. For the membrane with a ZPR structure, the local strain of sensor_X is almost constant under the same X-axis tensile strain and different Y-axis tensile strains, which indicates that Y-axis tensile strain has no effect on sensor_X. Similarly, the local strain on sensor_Y is also independent of X-axis tensile strain (Fig. S8). In contrast, for membranes without a ZPR structure, when the X-axis tensile strain is not changed, the local strain of sensor_X increases with an increase in Y-axis tensile strain, which indicates the clear interference caused by the Y-axis tensile strain. The comparison demonstrates that the ZPR sensor is capable of independent perception of biaxial strain. Moreover, scanning electron microcopy (SEM) images of sensing regions reveal the response of the ZPR sensor to uniaxial strain. As shown in Fig. 2e, the sensing area is divided into two parts. The rough parts correspond to grooves formed by laser engraving, and the smooth parts correspond to flat surfaces between the grooves, which were engraved by low laser power. Before uniaxial stretching, the gold thin film on the surface of two parts is intact. After X-axis uniaxial stretching, cracks appear at the junction of two parts, which is the strain concentration area (Fig. 2f). The side view of the rough part shows that the strain concentration structure has arc-shaped profiles (Fig. 2g).

### Sensing performance of the ZPR sensor

**Figure 3.**
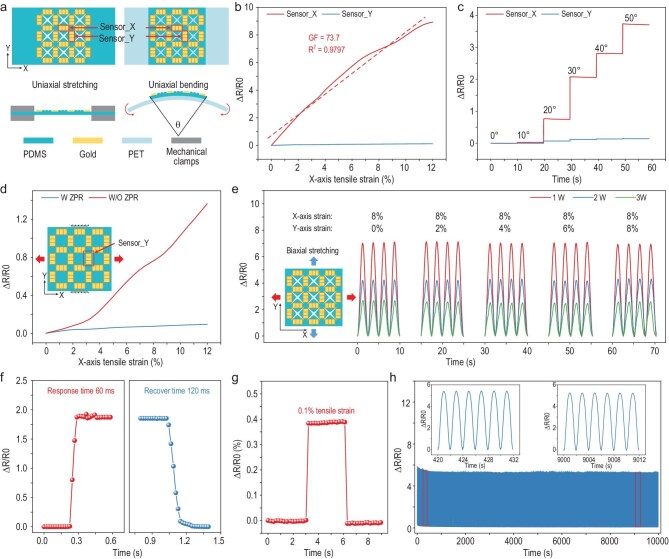
Measurement performance of ZPR sensors under different external stimuli. (a) Schematic diagrams of the ZPR sensor under uniaxial stretching and uniaxial bending. (b) Relative resistance response of X-axis and Y-axis sensing units under uniaxial X-axis tensile strain. (c) Relative resistance response of both axes’ sensing units to different uniaxial bending angles. (d) Relative resistance response of the Y-axis sensing unit on membranes with and without a ZPR structure under increasing X-axis tensile strain and 0% Y-axis tensile strain. (e) Resistance response of the X-axis sensing unit on membranes, to different biaxial strains. The different lines represent different membranes prepared by the molds engraved by different laser powers (1 W, 2 W, 3 W). (f) Instant response of the ZPR sensor under tensile strain loading and unloading processes. (g) Resistance response of the ZPR sensor under 0.1% tensile strain. (h) Reproducibility test of the ZPR sensor during 5000 loading-unloading cycles with 6% uniaxial tensile strain.

Due to its exotic mechanical properties, the ZPR structure can prevent the deformation of sensing units in a particular axis under stimuli in the perpendicular axis, ensuring the independent perception of uniaxial stimuli. Thus, two orthogonal sensing units in the ZPR sensor are able to independently detect biaxial stimuli, namely sensor_X and sensor_Y, as shown in Fig. 3a. To evaluate the performance of the ZPR sensor in response to uniaxial stimuli, uniaxial stretching and bending were applied, respectively, as shown in Fig. 3a. The uniaxial strain was applied to the sensor by clamping the excessive PDMS on the X-axis and stretching the entire membrane. For uniaxial bending stimuli, the sensor was attached to the smooth surface of a polyethylene terephthalate (PET) membrane, and a specific bending angle of the sensor was created by moving the edges of the flexible PET membrane. Notably, the moving edges were parallel to the Y-axis, meaning that the sensor was only bent around the Y-axis. The relative resistance changes of different sensing units in the ZPR sensor were recorded. The relative resistance change is defined as Δ*R*/*R*_0_, where Δ*R* is the resistance change under different stimuli, and *R*_0_ is the original resistance of the flexible sensor. The sensitivity of a flexible sensor, defined as the gauge factor (GF), is described as GF = Δ*R*/(*R*_0_*ε), where *ε* represents the applied strain. The relative resistance of sensor_X shows an almost linear increase against progressively increased X-axis tensile strain in the range of 0–12%, with R^2^ = 0.9797 and GF = 73.7. Meanwhile, the relative resistance of sensor_Y exhibits little increase regardless of the X-axis tensile strain (Fig. 3b). This significantly contrasting result demonstrates that the sensing units in a particular axis only respond to tensile strain in the corresponding axis. To investigate the stability and repeatability of a single sensing unit, seven loading-unloading cycles at a dynamic X-axis uniaxial tensile strain of 2%, 4%, 6% and 8% were applied to the sensor. Sensor_X exhibits highly repeatable responses at each applied strain over all the cycles (Fig. S9). For different tensile strains, the response curves are similar to each other, indicating stable and repeatable measurement performance. Resistance response of the sensor to uniaxial bending stimuli was also measured. As the bending angle increases from 0° to 50°, the relative resistance of sensor_X gradually increases (Fig. 3c). At the same time, little change in the resistance of sensor_Y is observed, demonstrating that the sensor can accurately distinguish the bending stimulus around the X-axis or Y-axis without any interference. Moreover, response comparisons of sensor_Y in membranes with and without a ZPR structure under increasing X-axis tensile strain and 0% Y-axis tensile strain are shown in Fig. 3d. The response of sensor_Y without ZPR increases with X-axis tensile strain, while the response of ZPR sensors exhibits little increase.

The previous FEA has already revealed that the independent response of the sensor to biaxial stimuli mainly originates from the ZPR structure, while the strain concentration structure contributed little to biaxial strain perception capability. Nevertheless, the sensitivity of the ZPR sensor was significantly enhanced by the strain concentration. To analyze the effect on sensing sensitivity, three different laser powers (1 W, 2 W, 3 W) were utilized to carve acrylic mold to achieve the preparation of a strain concentration structure with different thicknesses. High engraving power promoted deep grooves on the acrylic mold, resulting in a thick strain concentration structure. To eliminate the interference of other aspects, other ZPR structure parameters of three membranes were controlled to be the same. Under 10% X-axis tensile strain, the local strain of sensor_X decreases with the increase in engraving power, indicating that a thick strain concentration structure induces small local strain under the same tensile strain (Fig. S10). In contrast, the membrane with thick strain concentration structure fractured under low tensile force due to the high local strain on the sharp corners (Fig. S11). Moreover, three membranes were sputtered with gold by the same parameters to eliminate the influence of conductive materials. Relative resistance changes of sensor_X in three sensors over four loading-unloading cycles under different biaxial strains are shown in Fig. 3e. The X-axis tensile strain was constant at 8%, while the Y-axis tensile strain was 0%, 2%, 4%, 6% and 8%, respectively. Each sensor maintains approximately the same peak value throughout all loading cycles with different Y-axis tensile strains, confirming that the independent perception of biaxial strain is not affected by the parameter of strain concentration structure. Specifically, the relative resistance changes of the sensor prepared with high engraving power are lower than that of the sensor prepared with low engraving power regardless of the value of Y-axis tensile strain. The result demonstrates that a thick strain concentration structure leads to low sensitivity, which is consistent with the FEA results of the local strain.

Fast response, low measurement threshold and durability are also important for flexible sensors. As shown in Fig. 3f, the sensor demonstrates a fast response time of 60 ms during the loading process and a recovery time of 120 ms during the unloading process. In particular, a tensile strain as low as 0.1% is precisely detected, indicating that the sensor has great prospects in detecting tiny motions and vibrations (Fig. 3g). Besides, the resistance changes of the sensor under 5000 loading-unloading cycles at 6% uniaxial tensile strain (Fig. 3h), 6% X-axial and Y-axial tensile strain (Fig. S12), and 30° bending (Fig. S13) were invariable, indicating the remarkable durability and repeatability of the ZPR sensor.

### Detection of grasping force for objects with variable curvature

Successful and secure manipulations of deformable and fragile objects are quite challenging for rigid manipulators. For instance, excessive operating force may induce irreversible deformation of soft objects and break fragile objects, whereas low operating force cannot stabilize the grasp and causes slippage, which may lead to damage to both the objects and the manipulator. Consequently, accurate detection of grasping force is essential for manipulation. However, the signal of traditional flexible sensors attached to the surface of deformable objects will be affected by the changing curvature, which may cause incorrect grasping force and damage to objects.

**Figure 4.**
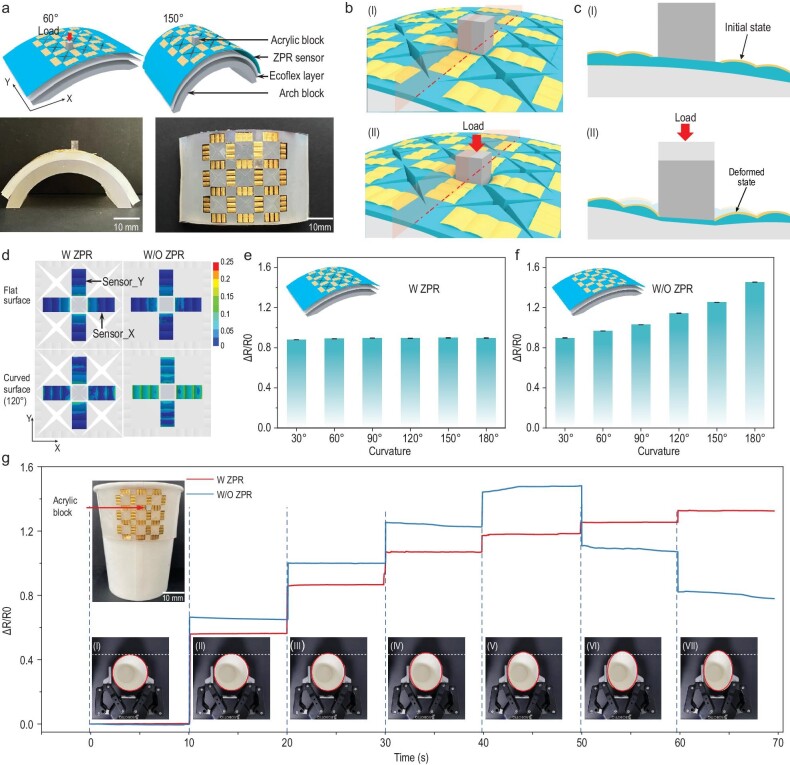
Detection of grasping force for objects with variable curvature. (a) Schematic diagrams of the ZPR sensor attached on different surfaces with 60° and 150° curvature (top), and photographs of the ZPR sensor attached on the surface with 150° curvature (bottom). (b) Schematic diagrams of the ZPR sensor before (top) and after (bottom) the loading of external force. (c) Cross-sectional diagrams of planes shown in (b) showing sensor_Y and the Ecoflex layer before (top) and after (bottom) the loading of external force. (d) Local strain contour maps of sensing units in membranes with (left) and without (right) a ZPR structure, when the external force is applied to the top surface of the acrylic block and the membranes are attached to a flat surface (top) and 120° curved surface (bottom). (e and f) Resistance response of sensor_Y in ZPR sensors (e) and sensors without a ZPR structure (f). Both the sensors are attached on surfaces with different curvatures, and the external force is constant at 1 N. (g) Response of sensor_Y in ZPR sensors and sensors without a ZPR structure during the paper-cup-grasping task. The top inset illustrates the ZPR sensor attached on the cup. The bottom photographs (from I to VII) illustrate a series of processes for increasing grasping force.

As the signals of sensor_Y with a ZPR structure are not affected by the bending stimuli around the Y-axis, the change in surface curvature of deformable objects does not affect sensor_Y, which ensures the accurate detection of grasping force. To further illustrate this characteristic, six arch blocks with different curvatures of 30°, 60°, 90°, 120°, 150° and 180°, were designed to estimate the pressure perception performance of ZPR sensors when attached onto surfaces with different curvatures. As shown in Fig. 4a, an additional layer of Ecoflex is inserted between the ZPR sensor and the arch block, and a square acrylic block is pasted next to sensing units to convert external pressure stimuli into strain stimuli. Figure 4b and c illustrate the deformation of the ZPR sensor when external pressure is exerted to the top surface of the acrylic block, and the force was made consistent by a dynamometer. To confirm that the curvature has little effect on the pressure perception of the ZPR sensor, the local strain of sensing units around the acrylic block was analyzed by FEA. The external force was constant at 1 N, and the ZPR sensor was attached to the flat surface and curved surface with 120° curvature, respectively. As shown in Fig. 4d, FEA results indicate that sensor_Y in the ZPR sensor withstands almost the same local strain under these two loading situations, which are determined by only the external load. However, sensor_Y in the sensor without a ZPR structure exhibits large local strain when the sensor is attached to the 120° curved surface, indicating evident interference by the curvature. The resistance responses of sensors attached on surfaces with different curvatures are shown in Fig. 4e and f. For ZPR sensors attached on surfaces with different curvatures, the response of sensor_Y maintains almost the same amplitude under the same external pressure, demonstrating that the bending stimulus around the Y-axis has no effect on pressure perception (Fig. 4e). The resistance response of the sensor without a ZPR structure increases with the curvature, indicating that different bending stimuli dramatically interfere with pressure perception (Fig. 4f). For the sensor without a ZPR structure, large bending curvature induces large local strain on sensor_Y, resulting in more cracks on sensor_Y and large relative resistance response.

Furthermore, to demonstrate the significant value of ZPR sensors in the grasping process of deformable objects by rigid manipulators, the task of grasping paper cups was preprogrammed with the two-finger manipulator. As shown in the inset of Fig. 4g, the ZPR sensor and the Ecoflex layer are attached together on the side wall of a paper cup. A square acrylic block is attached next to the pressure sensing unit to convert the operating forces of the flat manipulator into strain stimuli. The grasping force was controlled by varying the closing degree of the two-finger manipulator; 0% closing degree meant that the manipulator was fully open, and 100% closure meant that the two fingers fully contacted. The bottom insets in Fig. 4g illustrate states of the paper cup under gradually increasing grasping force. Specifically, when the closure degree was 18%, the manipulator first contacted the acrylic block on the sensor, resulting in a low magnitude fluctuation in the resistance of sensor_Y. This 18% closure was regarded as the initial grasping state, and the closure increased by 3% increments at each stage until it reached 36%. Clearly, the curvature of the cup decreased with the increase in closure. The responses of sensor_Y on sensors with and without ZPR structures during the process were recorded, respectively. As shown in Fig. 4g, the resistance of sensor_Y with a ZPR structure increases gradually with an increase in grasping force. Due to the remarkable biaxial strain independent perception characteristic, curvature change of the cup during the process does not affect the resistance of sensor_Y, ensuring accurate perception of the increasing grasping force. On the other hand, resistance of the sensor without a ZPR structure increases gradually under low grasping force; however, the resistance decreases after the closure reaches 30%. The inconsistent changes in resistance response and grasping force indicate that curvature of the cup has significant impact on force perception by the sensors without a ZPR structure. The resistance of sensor_Y without a ZPR structure is determined by the synergy of increased pressure and decreased curvature. The increased pressure contributes to an increase in resistance, while the decreased curvature results in a decrease in resistance. The reduced distance between the two fingers of the manipulator caused the compression of the Ecoflex layer and deformation of the cup. When the manipulator was at a small degree of closure, the applied pressure and reduced distance between the two fingers were dominant on resistance change, resulting in an increase in resistance. However, when the manipulator reached a large degree of closure (>30%), the decreased curvature was dominant and led to a decrease in resistance. In contrast, the decreasing curvature of the paper cup was unable to interfere with the signal of sensor_Y in ZPR sensors, ensuring that the deformation of the paper cup hardly affected the resistance of sensor_Y. After eliminating the effect of cup deformation, the resistance of sensor_Y is only affected by the compression of the Ecoflex layer, which increases with grasping force. Therefore, the resistance of sensor_Y in ZPR sensors can accurately indicate the increase in grasping force. The comparison highlights the great potential of ZPR sensors in guiding the rigid manipulator to safely grasp deformable objects.

### Distinguishing normal operation and collision

The secure operation of robotic manipulators in diverse and uncertain working environments is essential for completing manipulation tasks accurately. Collisions during movement not only induce unstable operation, but also cause damage to the manipulator. Thus, the precise detection of collisions is critical. However, traditional sensors attached to multi-finger manipulators are typically utilized to monitor the bending status of fingers, and are not able to detect the occurrence of collisions. Meanwhile, for flexible sensors capable of detecting collisions, the bending of fingers may induce the deformation of sensors, leading to interference signals and inaccurate perception. Elimination of interference signals is of significance for accurate collision detection.

**Figure 5.**
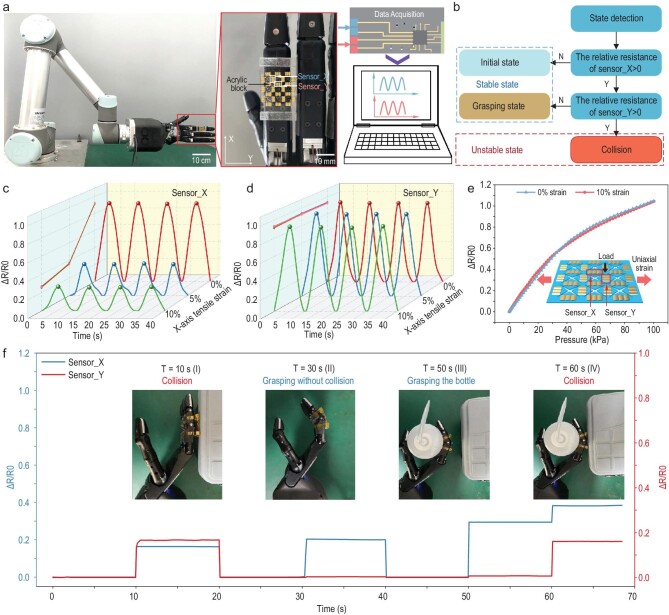
Detection of collisions during manipulation tasks. (a) Photographs of the bionic hand with the ZPR sensor and signal acquisition system. (b) Flow diagram of the strategy for distinguishing states of the manipulators. (c and d) Resistance response of X-axis (c) and Y-axis (d) sensing units on the ZPR sensor under the same external force of 1 N and different X-axis tensile strains (0%, 5% and 10%). (e) Response of the Y-axis sensing unit under 0% and 10% X-axis tensile strains to different external forces. (f) Response of X-axis and Y-axis sensing units in four states. The insets are photographs of actual states.

The ZPR sensor is not affected by the bending of fingers and can achieve accurate perception of collisions. As shown in Fig. 5a, the ZPR sensor is attached onto the forefinger joint of a bionic hand to detect different operation statuses. A square acrylic block is attached next to sensor_Y to convert collisions into strain stimuli. When the manipulator is in grasping state, the ZPR structure prevents sensor_Y from being affected by bending of the forefinger, while sensor_X, attached parallel to the bending direction of the forefinger, is stretched and the resistance increases. When collision occurs, the acrylic block is pressed, which induces tensile strain in both sensor_X and sensor_Y. Therefore, resistance of sensor_X changes both in grasping and collision states, while resistance of sensor_Y only changes in collision states. Based on this unique sensing performance, operating states of manipulators can be accurately detected by monitoring the resistance of sensor_X and sensor_Y. The detection procedures are illustrated in Fig. 5b. Since the resistance of sensor_X changes under both grasping and collision states, the unchanged resistance of sensor_X indicates that the manipulator is in the initial state. Once the resistance of sensor_X changes, the unchanged resistance of sensor_Y indicates that the manipulator is in grasping state, while the increase in resistance of sensor_Y indicates that a collision has occured. To demonstrate the role of sensor_X and sensor_Y in detecting the different operating states of the manipulators, resistance changes of sensor_X and sensor_Y on the ZPR sensor under pressure stimuli and uniaxial tensile strain (0%, 5%, 10%) were recorded. The pressure stimuli were introduced by utilizing the dynamometer to press the acrylic block, and the value of the external force was 1 N. The uniaxial tensile strain was introduced by stretching the ZPR sensor along the X-axis. As shown in Fig. 5c and d, the response of sensor_Y maintains almost the same amplitude, while the response of sensor_X decreases with an increase in tensile strain. The consistent response of sensor_Y is attributed to the unique mechanical properties of the ZPR structure, while the reduced response of sensor_X is due to the increase in original resistance. The X-axis stretching before the loading of pressure stimuli increased the resistance of sensor_X, resulting in large original resistance. Although the pressure stimulus was consistent, relative resistance decreased with an increase in original resistance. The relative resistance changes of sensor_Y under 0% and 10% X-axis tensile strain, and pressure stimulus from 0 kPa to 100 kPa, are exhibited in Fig. 5e. The response of sensor_Y under 0% and 10% X-axis tensile strain presents almost the same curve, demonstrating that the strain stimulus of the X-axis imposes little effect on the pressure perception of sensor_Y.

Four different operation states are demonstrated to confirm the accuracy of the ZPR sensor in detecting the status of manipulators. The resistance changes of sensor_X and sensor_Y during the whole manipulator task were recorded, as shown in Fig. 5f. Notably, collision occurred at 10 s and 60 s, while the states at 30 s and 50 s were normal grasping states by the manipulator. When the manipulator collided with the obstacle at 10 s, sensing units for both axes showed an increase in resistance. The manipulator was controlled to move away from the obstacle at 20 s, which induced the recovery of resistance for both the sensing units. When the forefinger was made to bend at 30 s and grasp a bottle at 50 s, the resistance of sensor_X increased, indicating the detection of bending states. The resistance of sensor_Y remained almost constant during these two states, demonstrating that the bending of fingers induced little interference to sensor_Y. However, when the manipulator collided with the obstacle again at 60 s, an increase in the resistance of sensor_Y was observed, indicating the accurate detection of a collision. Generally, the combination of two sensing units can effectively distinguish between the normal state and collision state of the manipulator, which is instrumental in ensuring safety in diverse manipulation tasks.

### Crawling direction and distance perception of biaxial soft robots

Steering motions are difficult to achieve with thermally actuated soft robots, which precludes their application in complex environments. Biaxial soft robots with the capability of independently walking along two perpendicular axes are a potential solution to tackle the challenge. For these biaxial soft robots, the deformation states of actuators are crucial for accurately estimating the locomotion status, which can maintain the correct walking direction and avoid collision with obstacles. However, conventional strain sensors attached to a particular actuator of a biaxial soft robot are always affected by actuation status in the perpendicular axis, which hinders the accurate perception of locomotion status.

**Figure 6.**
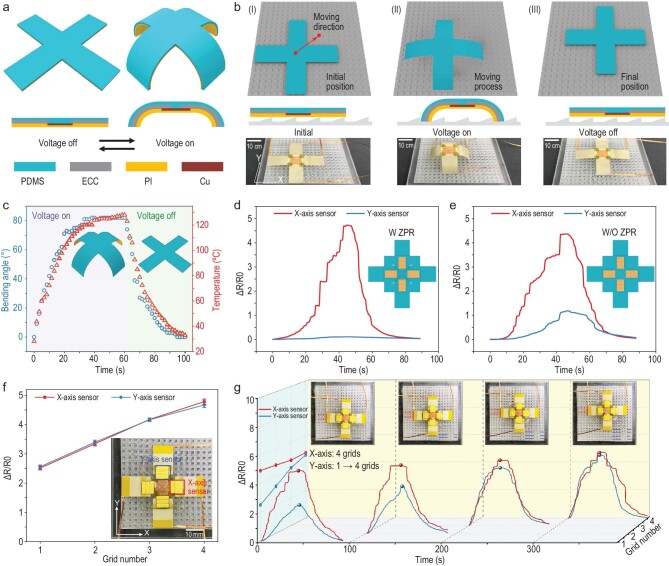
Detection of locomotion distance and direction of the biaxial crawling soft robot. (a) Schematic illustration of the biaxial soft robot during voltage on (right) and voltage off (left). The bottom schematic illustrates different layers of a single axis actuator. (b) Schematic illustrations (top and middle) and photographs (bottom) of the robot biaxial walking a distance of four grids in both the X-axis and Y-axis. Inset Ⅰ is the initial position and state of the robot, inset Ⅱ is the moving process and actuation state of the robot, and inset Ⅲ is the final position and state of the robot when the voltage is removed. (c) Curvature and temperature of a single leg as a function of time during actuation. (d and e) Response of the X-axis sensor on the membrane with (d) and without (e) a ZPR structure under X-axis actuation. Both the membranes are attached to the same soft robot. (f) Resistance response of both axes’ sensors to different crawling distances. (g) Response of sensors to constant X-axis locomotion distance and different Y-axis locomotion distances.

Due to the independent perception of biaxial stimuli, ZPR sensors are able to monitor the deformation states of actuators in different axes without interference. To illustrate the capability of the ZPR sensor when it comes to monitoring the deformation of soft robots, a biaxial crawling soft robot was prepared to perform diverse locomotion tasks. To achieve different locomotion statuses on different axes, the robot was designed as a cross-shaped film with four legs, and the actuation of each leg was realized by the deformation mismatch of two materials with different thermal expansion coefficients upon temperature rise. As shown in Fig. 6a, the biaxial soft robot is composed of a PDMS layer, an electrically conductive composite (ECC) layer and a polymide (PI) layer. The fabrication processes and detailed structure of the biaxial soft robot are illustrated in the supplementary data (Figs S14 and S15). To precisely control the locomotion distance of the robot, the acrylic plate with fish-scale protrusions was utilized as the platform for robot locomotion. Figure 6b illustrates the process of the robot moving along the X-axis. When voltage was only applied on the X-axis, the ECC layer of the two legs on the X-axis started to heat synchronously. Since the PDMS and PI layers had different thermal expansion coefficients, the heat drove each leg to bend towards the PI layer. Under continuous heating, the front leg tightly clasped the front side of a protrusion, which restricted the robot from moving backwards. The rear leg easily moved over one protrusion due to the smooth inclined surface. When the voltage was removed, the rear leg was stuck at the bottom of a protrusion, while the front leg moved over protrusions. Therefore, the moving distance of the soft robot was determined by the number of protrusions crossed by the rear leg during voltage loading. Optical photographs of the soft robot under a simultaneous actuation process on both axes are shown at the bottom of Fig. 6b.

To study the controllable movement of the soft robot during the actuation process in detail, the bending angle and temperature of the leg were monitored, with 3.5 V driving voltage (Fig. 6c). The temperature and bending angle increased with an increase in driving voltage (Fig. S16a), and the nearly linear correlation between bending angle and temperature change was determined by capturing optical photographs of a leg under different driving voltages (Fig. S16b). To accurately detect the bending angle of each leg and minimize the impact on actuator design, the sensor attached to the robot contains only one element of the ZPR structure, and the direction of strain concentration structure is also changed to adapt to the bending direction of actuators. When the voltage was only applied to the X-axis ECC layer, resistance of the sensing unit located on the X-axis legs increased dramatically with time, while the sensing unit on the Y-axis legs exhibited no response change (Fig. 6d), indicating the independent detection of single axis locomotion status. The responses of sensing units on the membrane without a ZPR structure under the same voltage condition were also recorded (Fig. 6e). For the sensor without a ZPR structure, the sensing unit on a Y-axis leg obtained an increased resistance when no voltage was applied to the Y-axis ECC layer, which suggested apparent interference by the locomotion of X-axis actuators. The responses were similar when the voltage was only applied on the Y-axis (Fig. S17).

The correlation between the sensor response and the locomotion distance was determined by recording the resistance changes as the robot crossed a different number of grids in the protrusion. Notably, the response of the X-axis (Y-axis) sensing unit was obtained when the voltage was only applied on the X-axis (Y-axis). The response curves of the X-axis and Y-axis sensing units are shown in Fig. 6f, and the optical photographs of the sensing units, actuator and locomotion platform are shown in the inset. Both the sensing units exhibited increased resistance with an increase in the number of grids across. The resistance response curves of the X-axis sensing unit under different X-axis uniaxial moving distances (one to four grids) are shown in the supplementary data (Fig. S18). Additionally, four different walking tasks with the same X-axis locomotion distance (four grids) and different Y-axis locomotion distances (from one grid to four grids) were performed to evaluate the performance of the sensor under biaxial simultaneous actuation. The different Y-axis locomotion distance was achieved by applying different voltage (2 V, 2.5 V, 3 V and 3.5 V) to the Y-axis ECC layers, while the voltage simultaneously applied on the X-axis ECC layers was consistent (3.5 V). As shown in Fig. 6g, the relative resistance change of the X-axis sensing unit remains at almost the same peak value during four walking tasks, while the response of the Y-axis sensing unit increases with the number of grids across. The movie of the robot crawling four grids along the X-axis and two grids along the Y-axis can be found in the supplementary data (Movie S2). The independent performance of sensing units on the X-axis and Y-axis during biaxial actuation confirms the ability of the sensor to monitor the locomotion direction and distance of soft robots, which promotes the integration of perception and actuation.

## CONCLUSION

In summary, we have presented a generic strategy to alter the Poisson's ratio of mechanical metamaterials, and developed flexible sensors with ZPR. The metamaterial membrane can demonstrate different Poisson's ratios via the elaborate design of topological parameters and arrangement of the PPR structure and the typically NPR structure. The membrane is optimized to realize near-ZPR, which enables a constant transverse width under longitudinal stretching. Due to the counterintuitive mechanical properties, uniaxial tensile strain induces little strain in the sensing units of the perpendicular axis, which endows the sensors with the capability of independent detection of biaxial stimuli. Strain concentration structures embedded on sensing regions to control the location of cracks further eliminate interference and stabilize the resistance response. The biaxial sensors are capable of perceiving biaxial tensile strain from 0% to 12% on both axes without any interference.

We demonstrate that the extraordinary characteristics of ZPR flexible sensors enable accurate force, strain and motion perception in cases of robotic manipulation and locomotion with complex deformation, while sensors without ZPR provide erroneous measurements. The sensors attached to deformable objects are able to guide rigid manipulators to safely grasp the object. The sensors attached on the fingers of the manipulator are capable of distinguishing the normal bending of fingers from unexpected collisions with obstacles. As well as applications in robotic tactile systems, the ZPR sensors are also able to detect the locomotion distance and direction of a biaxial soft robot, indicating their great potential for application in proprioceptive soft robots. Continuous improvements focusing on reducing structural dimensions and expanding sensing range are underway to expand the applications of the sensors.

## METHODS

### Fabrication of the ZPR sensor

A piece of 40 mm × 20 mm × 5 mm acrylic plate was cut by a carbon dioxide (CO_2_) laser cutter (Guangzhou HZZ E-Photo Technology Co. Ltd., ILS-3V). The three-dimensional model of the strain concentration structure was converted to bitmap by the sculpture software. The bitmap containing structural parameters was sent to the laser-cutting machine to carve grooves on the surface of the acrylic plate. The laser power was 1 W and the laser movement speed 10%. Next, the star acrylic molds were obtained by laser cutting a piece of 40 mm × 20 mm × 0.2 mm acrylic plate based on the structural parameters, while the remaining thin acrylic plate was utilized as the locator of the star molds. After using adhesive to stick the star molds onto the previously prepared thick acrylic plate, PDMS (Dow Corning Sylgard 184, a 10 : 1 mixture of silicone prepolymer and curing agent) was poured into the whole mold and spin coated at 300 r/min for 30 s. After curing in an 80°C oven for 2 h, the PDMS membrane with a ZPR structure and strain concentration structure was cut to 40 mm × 17.6 mm and peeled off from the mold, while the excess PDMS was reversed for fixture clamping. Then, a PET film hollowed out in the corresponding area of the strain concentration structure was pasted onto the surface of the PDMS membrane. Gold was sputtered onto the surface of the whole film by magnetron sputtering (Chinese Academy of Sciences Shenyang Scientific Instrument Co., Ltd., TRP-450).

### Fabrication of the crawling soft robot

10% polyvinyl alcohol (PVA) aqueous solution was spin coated onto a glass wafer at 1000 rpm for 30 s and dried at 90°C for 30 min to form the sacrificial layer. The wallpaper with four hollowed areas corresponding to the legs was stuck on the glass wafer. The ECCs, prepared by the process mentioned before [53], filled the hollow area and were scraped flat with a blade. After peeling off the wallpaper, the glass wafer was put into a 160°C oven for 1.5 h to cure the ECC layer. Then, the PDMS was spin coated on the ECC layer at 300 rpm for 30 s, followed by curing at 80°C for 2 h. After cutting off the excess PDMS, the cross-shaped sample was placed in deionized water at 80°C for 8 h to remove the sacrificial layer. Afterward, a copper foil was connected to two legs in the same axis by silver paste, and PI tape (Kapton, DuPont) was pasted on the surface of the connected copper foil to prevent electrical connections between different axes. Four copper foils were connected to the edges of four ECC areas by silver paste. Finally, the commercial self-adhesive PI tape was pasted on the surface of the ECC layers to fabricate the soft robot with PDMS/ECC/PI actuators.

### Characterizations of morphology

The SEM images of the sensor were captured by a Hitachi SU3900 (15 kV). The morphology of a single axis actuator under applied voltage was captured by a Sony ILCE-7M2, and the photos were imported into AutoCAD to extract the bending angle. The temperature of the actuator was measured by an infrared camera (FLIR T1050sc), which was placed 20 cm above the actuator.

### Characterizations of the mechanical properties

The stress-strain curves of the ZPR membrane were measured and recorded by a computer-controlled testing machine (Instron-5942), and a strain rate of 60 um s^−1^ was applied during stretching. The Poisson's ratio of the membrane was calculated by the formula ${\mathrm{v\ = \ }}\frac{{\Delta {\mathrm{X}}/{\mathrm{X0}}}}{{\Delta {\mathrm{L}}/{\mathrm{L0}}}}$, which was simplified to v = ΔX/ΔL due to having the same initial transverse and longitudinal length. The longitudinal deformation of the boundary area corresponding to the left/right star-hollowed column was regarded as the longitudinal deformation of the overall membrane ΔX. The longitudinal deformation of the ZPR structure in FEA was measured based on the deformation curve (Figs S1 and S2). The longitudinal deformation of the ZPR membrane in experiments was measured by analyzing the digital photographs, which were captured under increasing transverse tensile strain by a camera (Sony ILCE-7M2).

### Characterizations of the measurement performance of the sensor

Both uniaxial and biaxial tensile strains were applied using a biaxial tensile testing platform (FlexTest Mini S2-P, Hunan NanoUp Electronics Technology Co., Ltd.). The external pressure was applied and measured by a computer-controlled Z-stage with a force gauge (HP-5N, HandPai). The resistances of sensor units were measured by a digital multimeter (Tektronix DMM6500) with a multichannel scanning module (Tektronix 2000-SCAN). The raw data were recorded in the registers of the digital multimeter and then transmitted to a computer for analysis.

### Grasping and manipulation tests

The grasping task was performed by a robotic gripper (2FINGER-85, ROBOTIQ). The manipulation task was performed by a bionic hand (SHADOW DEXTEROUS HAND LITE, Shadow Robot Company). Both of the manipulators were mounted on a robot arm (UR5, UNIVERSAL ROBOTS) to perform operation tasks. The robotic gripper was controlled by the terminal of the robot arm, while the bionic hand was controlled by a computer with an robot operating system.

## Supplementary Material

nwae027_Supplemental_Files

## Data Availability

All data are presented in the paper or in the Supplementary Data. All information and materials can be requested from the corresponding author.
