# Supplementary material for: Flexible sensors with zero Poisson's ratio
Source: Natl Sci Rev. 2024 Jan 23;11(5):nwae027. doi: 10.1093/nsr/nwae027 (PMC10989663; doi:10.1093/nsr/nwae027)
Supplement: nwae027_Supplemental_Files [file nwae027_supplemental_files.zip › 20231124 - Supplementary materials - revision.pdf]

Supplementary Materials for

Flexible Sensors with Zero Poisson's Ratio

Xin Huang[1], Tianzhao Bu[1], Qingyang Zheng[1], Shaoyu Liu[1], Yangyang Li[1], Han Fang[1], Yuqi Qiu[1], Bin Xie[1], Zhouping Yin[1], Hao Wu[1,2,*]

[1]Flexible Electronics Research Center, State Key Laboratory of Intelligent Manufacturing Equipment and Technology, School of Mechanical Science and Engineering, Huazhong University of Science and Technology, Wuhan, Hubei, 430074, China.
[2]School of Integrated Circuits, Huazhong University of Science and Technology, Wuhan, Hubei, 430074, China.
*Corresponding author. Email: hwu16@hust.edu.cn

This PDF file includes:

Supplementary text

Figure S1 to S18

Other Supplementary Material for this manuscript includes the following:

Movie S1 (.mp4 format). Comparison of PDMS membranes with and without ZPR structure.
Movie S2 (.mp4 format). Biaxial actuation of the soft robot.

Supplementary Text

Calculation of deformation and strain distribution of the ZPR membrane

The Static Structural module in the commercial software ANSYS Workbench (V18.0) was utilized to study the deformation and stain distribution of the ZPR membrane under different tensile strain. The dimension parameters of the ZPR membrane are shown in Fig. 1B. The thickness of the ZPR membrane was 200 μm. PDMS with elastic modulus of 1.62 MPa and Poisson's ratio of 0.49 was selected as the constitute material of the ZPR membrane. The tetrahedral element was chosen to mesh the 3D model of the ZPR membrane, and the element size was controlled to be 0.2 mm.

The tensile strain was applied on the side of the ZPR membrane. Since the ZPR membrane was fixed on the testing platform during tensile testing, the side of the ZPR membrane was constant in Z-axis. Specifically, for X-axis uniaxial stretching analysis, the side perpendicular to X-axis was applied with tensile strain along X-axis, zero displacement along Y-axis and zero displacement along Z-axis, while the side parallel to X-axis was free. For biaxial stretching analysis, the side perpendicular to X-axis was applied with tensile strain along X-axis, free in Y-axis and zero displacement along Z-axis, while the side parallel to X-axis was applied with tensile strain along Y-axis, free in X-axis and zero displacement along Z-axis.

Calculation of bending angle of the biaxial soft robot

The bending angle of single leg of the biaxial soft robot under different temperature change was calculated by the Steady-State Thermal module and the Static Structural module in the commercial software ANSYS Workbench (V18.0). The Steady-State Thermal module calculated the temperature of the leg under different temperature of the ECC layer. The Static Structural calculated the deformation caused by temperature under boundary conditions. The detailed structure of the biaxial soft robot is illustrated in Fig. S14B. The biaxial soft robot was composed of the PDMS layer, the PI layer, the ECC layer and two copper foils. The thickness of the PDMS layer was 200 μm, the PI layer, the ECC layer and the copper foils were 100 μm thick. The following materials properties were defined in the model: the PDMS layer (elastic modulus 1.62 MPa,

Poisson's ratio 0.49, coefficient of thermal expansion $3.1e^{-4}$ K^{-1} and thermal conductivity 0.147 W/(m×k)), the PI layer (elastic modulus 170 MPa, Poisson's ratio 0.38, coefficient of thermal expansion $2e^{-5}$ K^{-1} and thermal conductivity 0.25 W/(m×k)), the ECC layer (simplified to PDMS) and the copper foil (elastic modulus $1.18e^{5}$ MPa, Poisson's ratio 0.34, coefficient of thermal expansion $1.65e^{-5}$ K^{-1} and thermal conductivity 401 W/(m×k)).

The ambient temperature was defined as 25°C, the temperature of the ECC layer gradually increased from 25°C to 135°C, corresponding to the temperature change from 0°C to 110°C. Since the side perpendicular to X-axis always contacted with the platform during actuation process of the leg, the side was fixed in Y-axis and Z-axis, and free in X-axis.

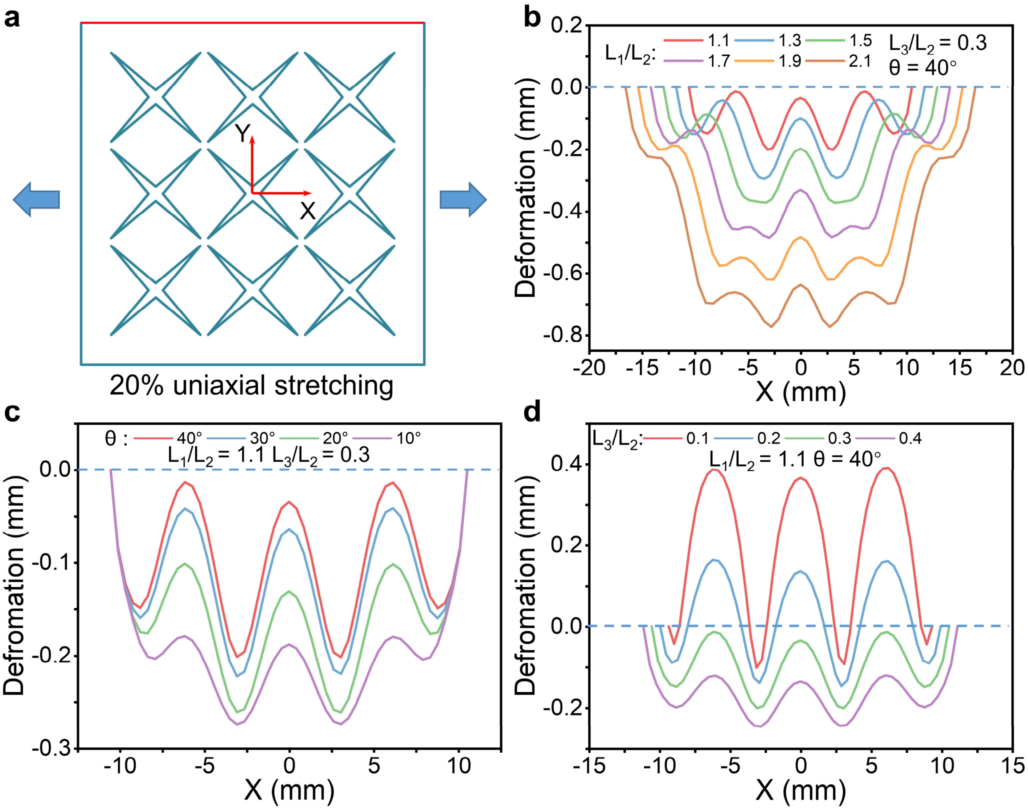

Figure S1. Longitudinal deformation of the top boundary of the zero Poisson's ratio (ZPR) structure under 20% lateral tension. The tensile strain was applied on the side perpendicular to X-axis, and the side parallel to X-axis was free. **(a)** Schematic diagram of the ZPR structure and the upper boundary. **(b-d)** Longitudinal deformation of the upper boundary on the membranes with different L_1 (b), θ (c) and L_3 (d). These results obtained by finite element analysis (FEA) indicates morphology of the top boundary of the ZPR structure after 20% stretching. The dashed line represents the location of the initial boundary. The curve representing the deformed boundary of the ZPR structure ($L_1 = 5$ mm, $L_3 = 1.5$ mm, $\theta = 40^o$) is closed to the dashed line, indicating the Poisson's ratio of the ZPR structure is close to zero. Notably, when L_3 was smaller than 1.5 mm, the top boundary was convex, indicating negative Poisson's ratio of the structure.

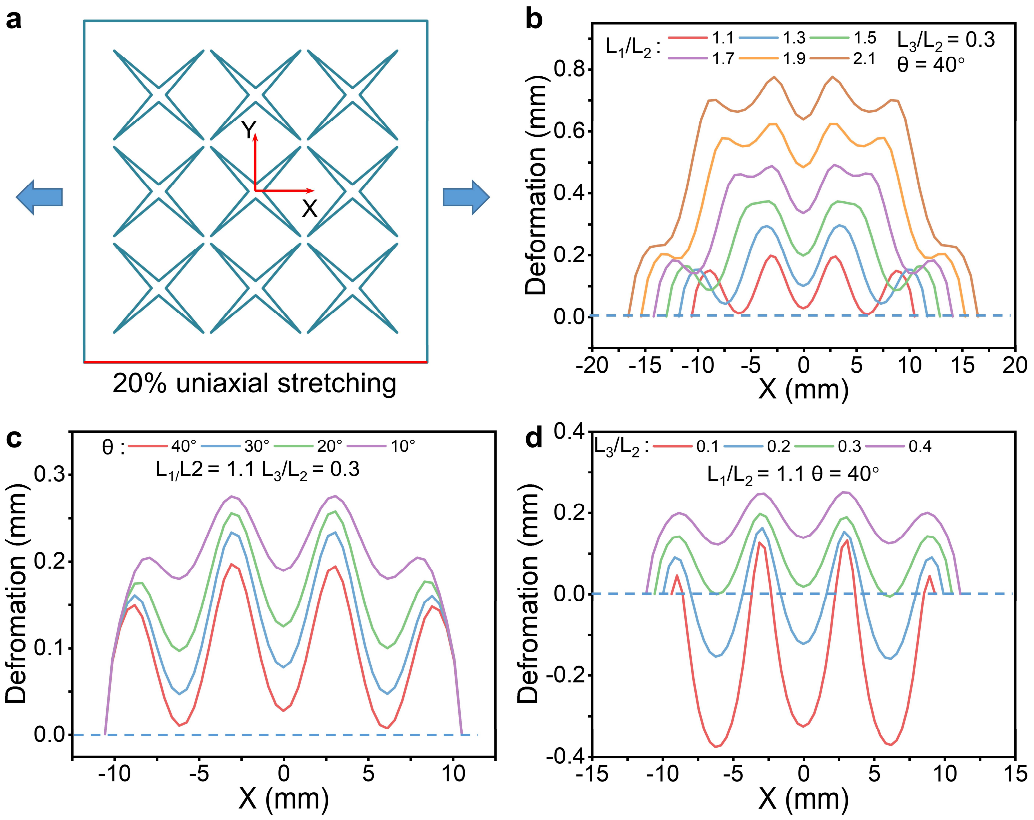

Figure S2. Longitudinal deformation of the bottom boundary of the ZPR structure under 20% lateral tension. The tensile strain was applied on the side perpendicular to X-axis, and the side parallel to X-axis was free. **(a)** Schematic diagram of the ZPR structure and the lower boundary. **(b-d)** Longitudinal deformation of the lower boundary on the membranes with different L_1 (b), θ (c) and L_3 (d). The dashed line indicates the zero longitudinal deformation. These results were also obtained by FEA, and symmetric with the results of the top boundary.

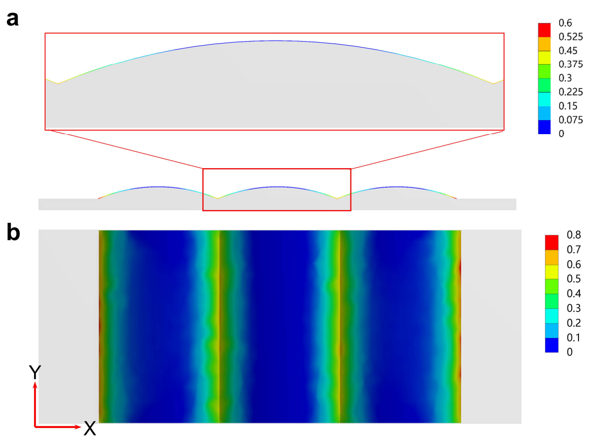

Figure S3. Strain contour maps of strain concentration structure under 20% tensile strain. The strain was applied along X-axis. (a) Strain contour map of the boundary of the strain concentration structure. **(b)** Strain contour of the surface of the strain concentration structure. These results show that large local strain is induced in the valley area of the strain concentration structure, while the peak area has small local strain. The maximum local strain of the boundary of the strain concentration structure occurs at the intersection of the arc-shape profiles, which is three times of the applied strain. The strain concentration effect induces cracks to generate in the valley area rather than random areas, which contributes to stable sensing signals.

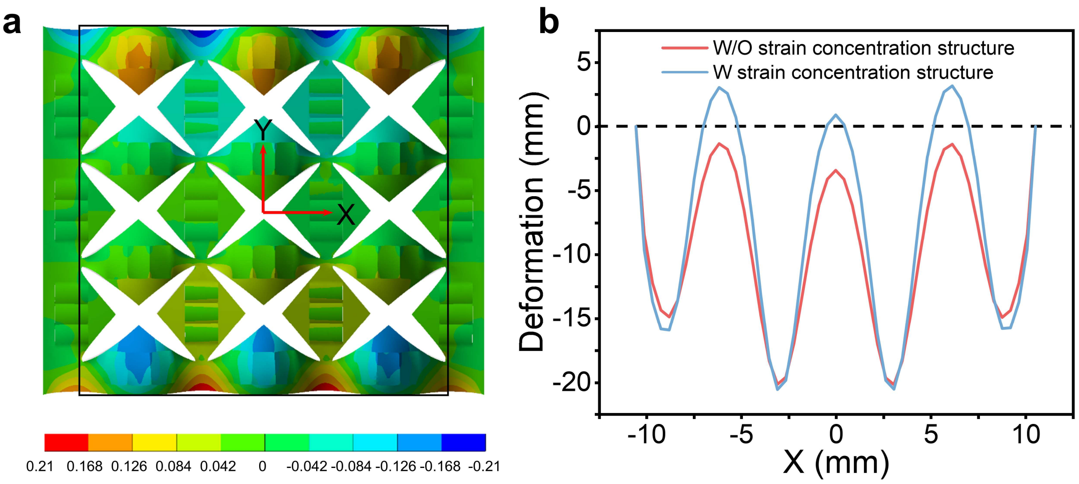

Figure S4. Comparisons of the ZPR membranes with and without strain concentration structures. 20% X-axis tensile strain was applied on the side parallel to Y-axis. (a) Longitudinal deformation contour map of the ZPR membrane with strain concentration structures. The black wireframe represents initial size of the membrane. **(b)** Longitudinal deformation of the top boundary of ZPR membranes with (blue) and without (red) strain concentration structure under 20% transverse tensile strain. The dashed line indicates the location of the initial top boundary. Although the top boundary of the ZPR membrane with strain concentration structure protrudes above the initial boundary, the value of deformation is similar with that of the boundary of the ZPR membrane without strain concentration structure, indicating the similar value of Poisson's ratio.

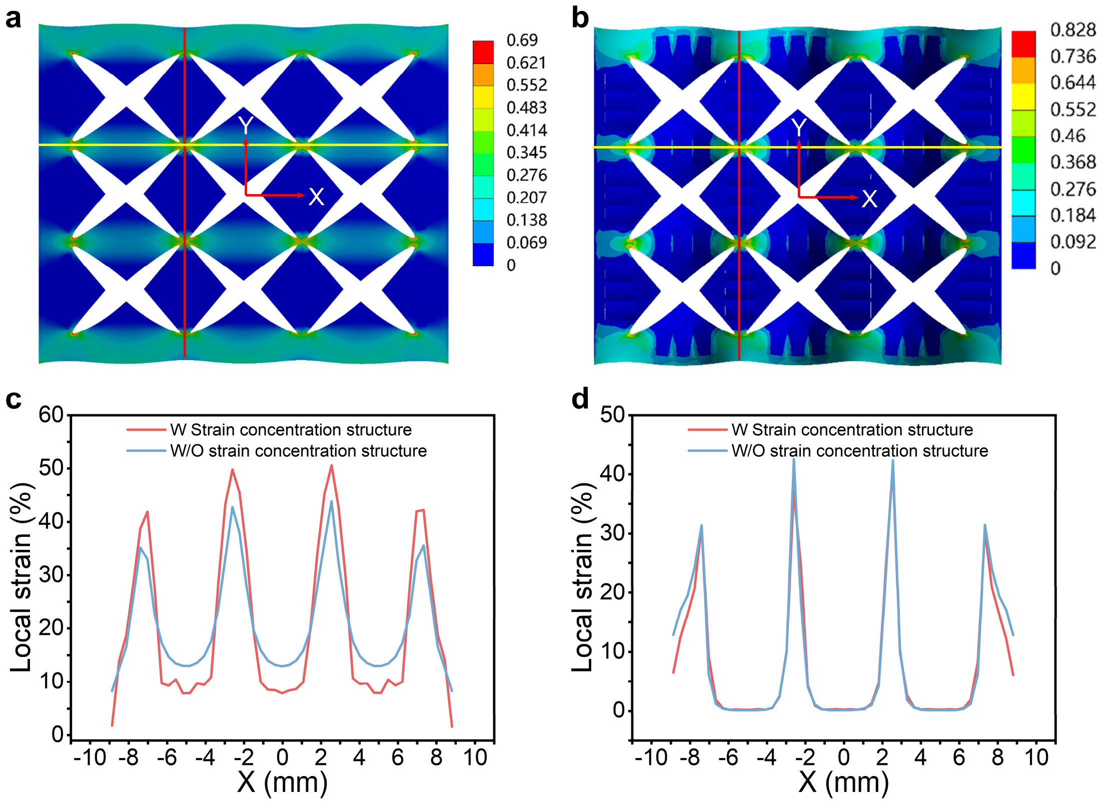

Figure S5. Strain distribution of ZPR membranes with and without strain concentration structure under 20% tensile strain in X-axis. (a) Strain contour map of the ZPR membrane without strain concentration structures under 20% X-axis tensile strain. **(b)** Strain contour map of the ZPR membrane with strain concentration structures under 20% X-axis tensile strain. **(c)** Local strain of the region highlighted by yellow line in (a) and (b). **(d)** Local strain of the region highlighted by red line red in (a) and (b). For the area parallel to X-axis (yellow line in (a) and (b)), the local strain of the sensing area in the ZPR membrane with strain concentration structure (red curve in (c)) is smaller than that in the ZPR membrane without strain concentration structure (blue curve in (c)), which contributes to extending sensing range of the ZPR sensors. For the area parallel to Y-axis (red line in (a) and (b)), the sensing areas in ZPR membranes with and without strain concentration structure both exhibit small local strain (nearly zero) under X-axis tensile strain, indicating the X-axis tensile strain has no effect on the Y-axis sensing area.

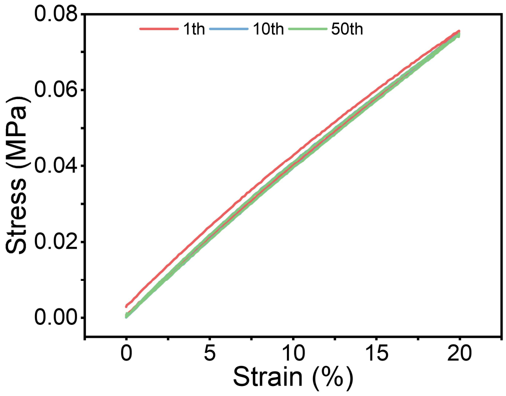

Figure S6. Tensile stress-strain curves of the metamaterial membranes with the ZPR structure and strain concentration structures. The result indicates that the elastic modulus of the ZPR membrane is 0.375 MPa. Compared with the flat PDMS membrane, the ZPR membrane has smaller stiffness.

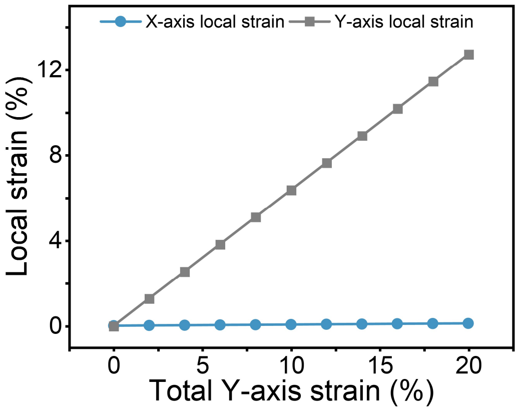

Figure S7. FEA results of the local strain of sensing units under different uniaxial Y-axis tensile strain. The Y-axis tensile strain gradually increased from 0% to 20%, and the X-axis was free of tensile strain. The Y-axis sensing units exhibits large local strain which increases with the applied strain, while the X-axis sensing units exhibits very low level of local strain under increasing Y-axis tensile strain, indicating that the ZPR structure can prevent Y-axis tensile from affecting X-axis sensing units.

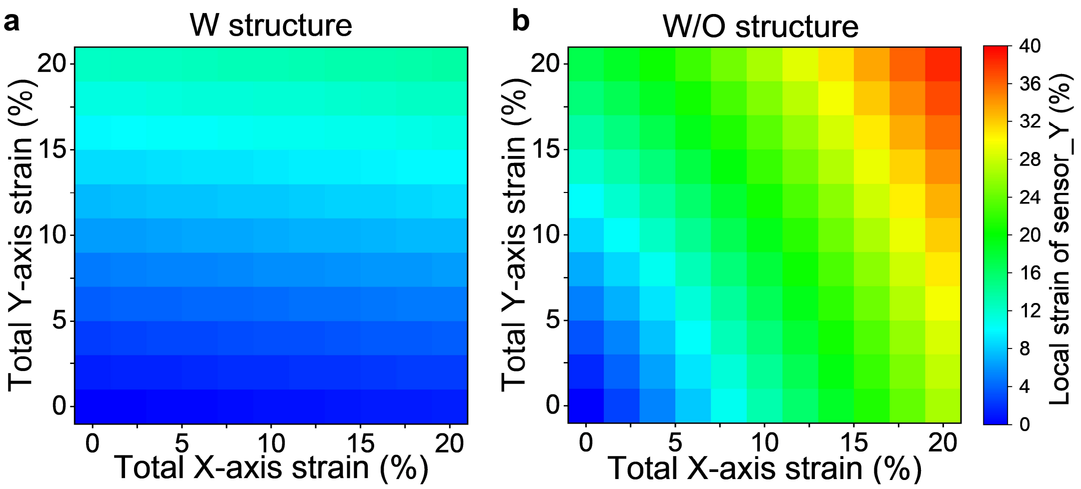

Figure S8. Local strain of the Y-axis sensing unit on membranes with ZPR structure and strain concentration structure (a), and only with strain concentration structure (b) under different biaxial strain. The hierarchical local strain contour map of the Y-axis sensing unit on the ZPR sensor indicates that the increasing X-axis tensile strain has no effect on the Y-axis sensing unit, which ensures that resistance change of the Y-axis sensing unit is only related to Y-axis tensile strain.

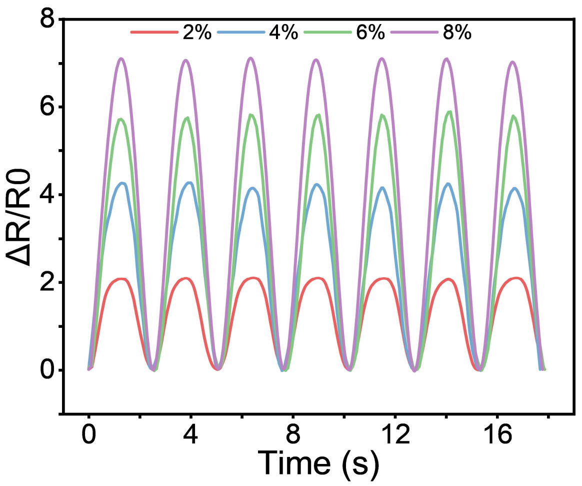

Figure S9. Repeated resistance response of the ZPR sensor to different tensile strain. The dynamic X-axis tensile strain of 2%, 4%, 6%, 8% were applied to the ZPR sensor, the relative resistance responses of the X-axis sensing unit were recorded to evaluate the stability and repeatability of a single sensing unit. The similar response curves indicate stable and repeatable sensing performance.

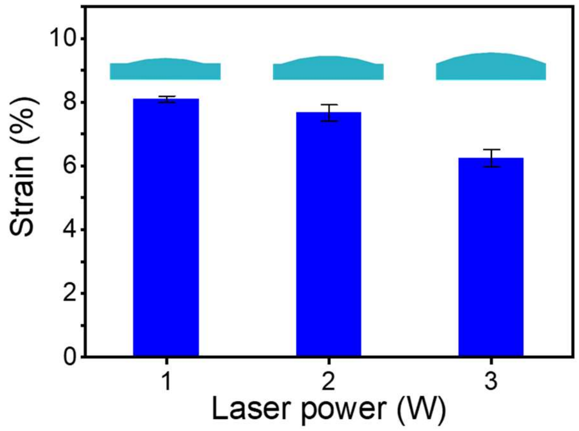

Figure S10. Local strain of the strain concentration area of the strain concentration structure with different thickness. 10% X-axis tensile strain was applied to the ZPR membrane, and the strain concentration structure was in the X-axis sensing unit. Different laser powers (1W, 2W, 3W) were utilized to carve acrylic mold to achieve the preparation of strain concentration structure with different thickness. High engraving power promoted deep grooves on the acrylic mold, resulting in thick strain concentration structure. The insert illustrates strain concentration structure with different thickness. Under 10% X-axis tensile strain, local strain of the strain concentration area decreased with the increase of engraving power, indicating that thick strain concentration structure induced small local strain under the same tensile strain. Therefore, sensing units with thick strain concentration structure exhibited small resistance change under the same tensile strain, and sensing units with thin strain concentration structure exhibited large resistance change and high sensitivity.

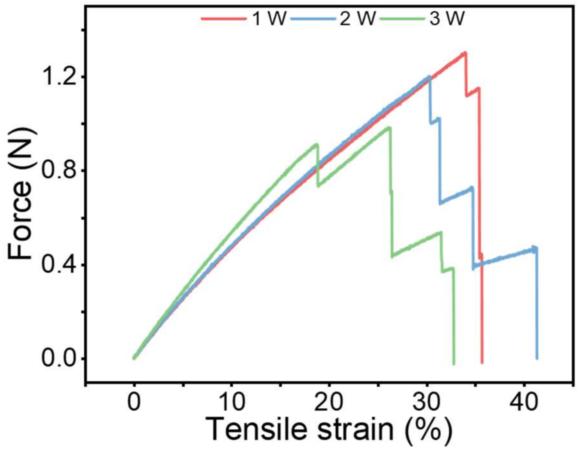

Figure S11. The tensile strain and tensile force of ZPR membranes with different strain concentration structures when stretched to fracture. The computer controlled testing machine (Instron-5942) was utilized to measure the tensile force and the tensile strain. When cracks occurred at the sharp corner of the membrane and propagated to fracture, the tensile force sharply decreased. After the first sharp corner was fractured, the local strain on other sharp corners reduced. Continuous stretching increased the local strain on sharp corners and caused fractures at other sharp corners. After all the sharp corners in the same row were broken, the ZPR membrane was fracture and the tensile force decreased to zero.

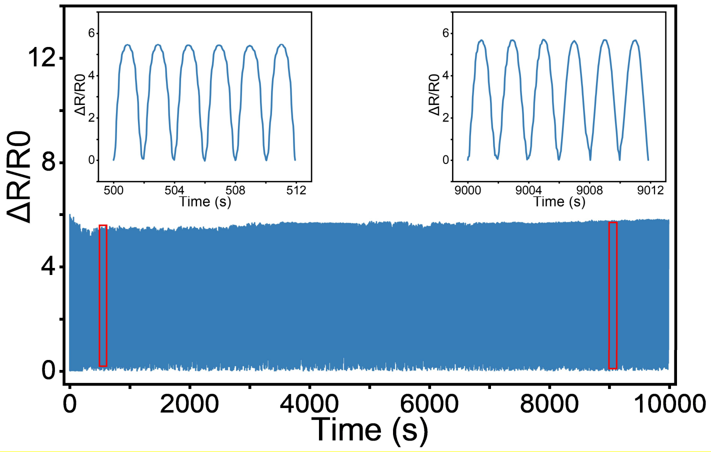

Figure S12. Reproducibility test of the ZPR sensor during 5000 loading-unloading cycles with 6% X-axis and Y-axis tensile strain. The stable responses with no drift indicate outstanding durability and repeatability of the ZPR sensor under biaxial tensile strain.

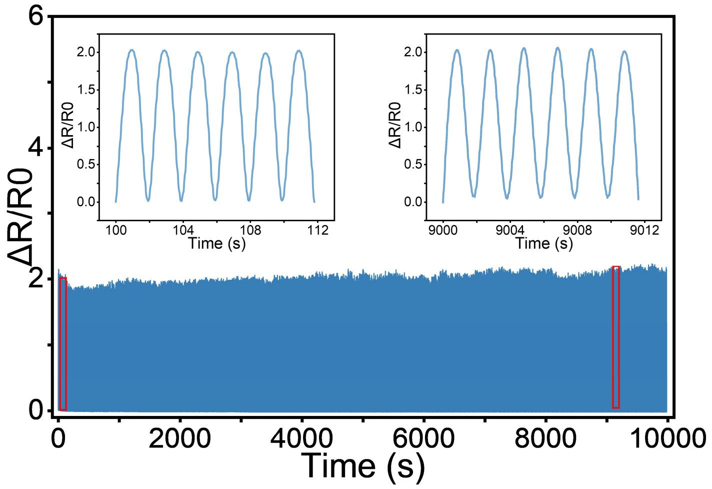

Figure S13. Reproducibility test of the ZPR sensor during 5000 loading-unloading cycles with 30° bending. The stable responses with no drift indicate outstanding durability and repeatability of the ZPR sensor under uniaxial bending stimuli.

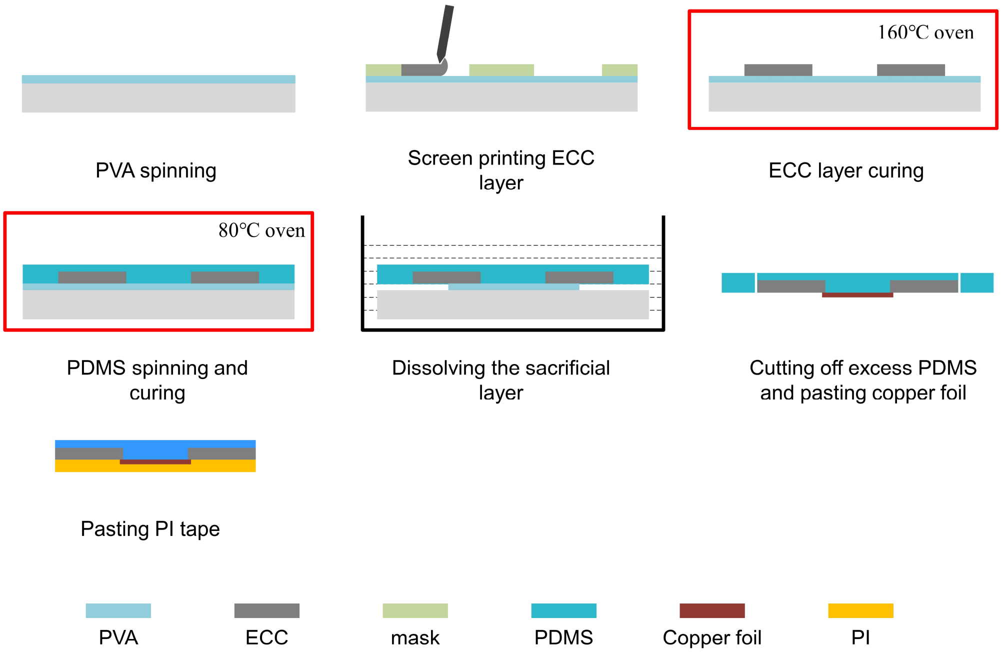

Figure S14. Schematic illustration of fabrication processes of the biaxial soft robot.

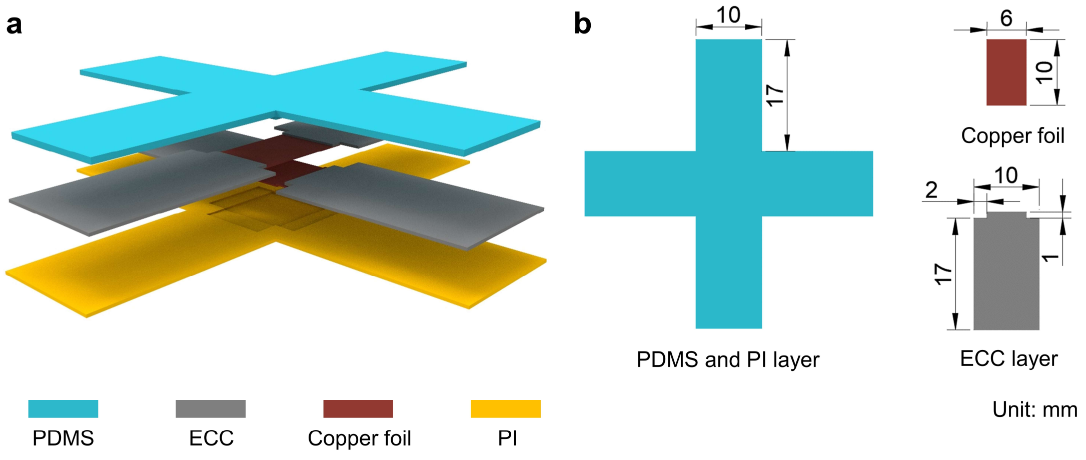

Figure S15. Schematic diagram of different layers of the biaxial soft robot. (a) Explosive view of the biaxial soft robot structure. The two legs on the same axis were electrically connected with copper foil, and the connecting copper foils of different axes were electrically separated by PI tape. **(b)** Geometric dimensions of different layers of the biaxial soft robot.

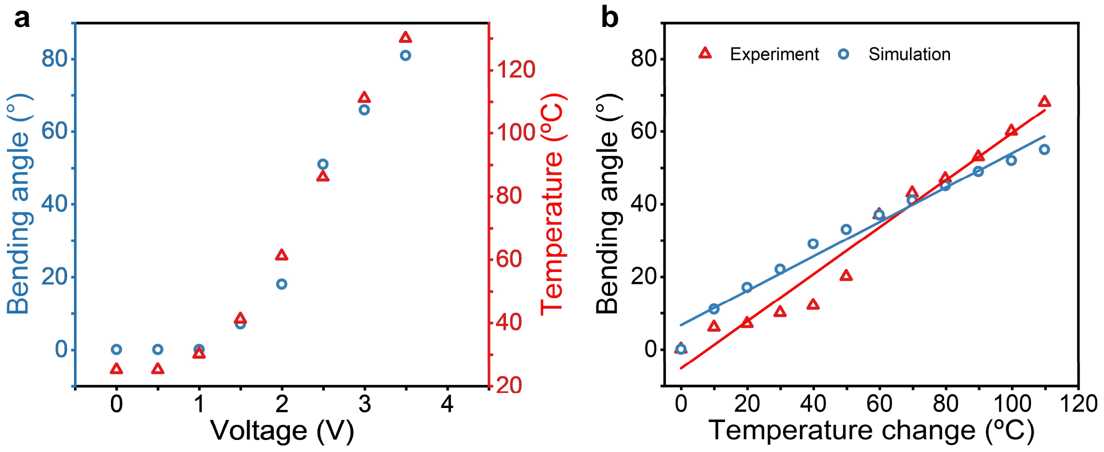

Figure S16. Actuation performance of a single leg of the biaxial soft robot. (a) Bending angle and temperature of the leg under different actuation voltage. The bending angle was measured by capturing the morphology of a single axis actuator when different voltage was applied. The temperature was measured by the infrared camera. **(b)** The experiment and simulation results of bending angle of the leg as a function of temperature change.

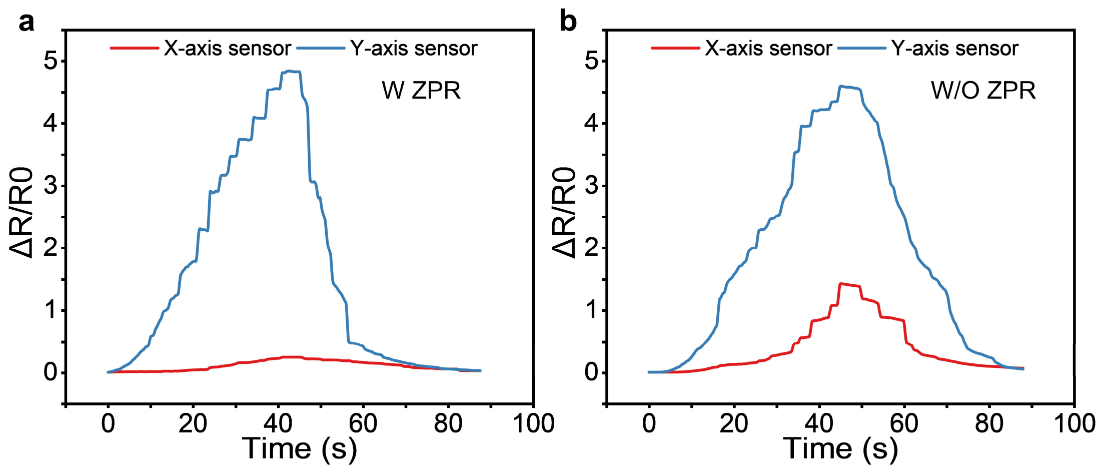

Figure S17. Response of the Y-axis sensor on the membranes with (a) and without (b) ZPR structure under Y-axis actuation. For the biaxial soft robot attached with the ZPR sensor, when the voltage was only applied to the Y-axis ECC layer, resistance of the sensing unit on Y-axis legs increased with time, while the sensing unit on X-axis legs exhibited little resistance change, indicating the independent detection of Y-axis locomotion status. For the biaxial soft robot attached with the sensor without ZPR structure, the sensing unit on X-axis legs exhibited an increased resistance when no voltage was applied on the X-axis ECC layer, which demonstrated an apparent interference of the Y-axis actuation. The comparison confirms the capability of the ZPR sensor in independently monitoring locomotion status of both axes, which cannot be achieved by the sensor without ZPR structure.

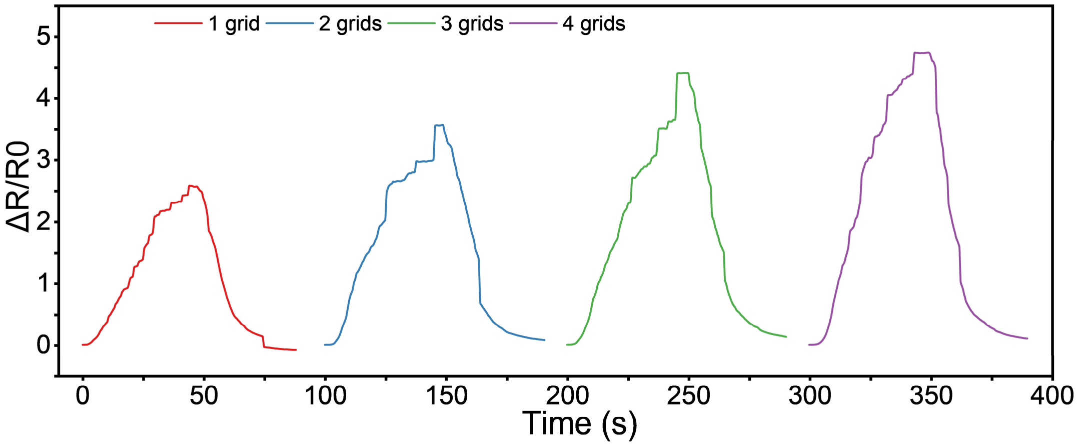

Figure S18. Resistance response of the X-axis sensing unit under uniaxial movement with different number of grids. Different voltage was applied to the X-axis ECC layer to actuate the X-axis legs to move different distance (from 1 grid to 4 grids). Resistance responses of the sensing unit on X-axis legs during the actuation processes were recorded. The increasing peak value with the number of grids indicates accurate detection of X-axis locomotion distance, demonstrating the capability of the ZPR sensor in detecting the single axis locomotion distance of the biaxial soft robot.
